# Integrated photonics with programmable non-volatile memory

**DOI:** 10.1038/srep22616

**Published:** 2016-03-04

**Authors:** Jun-Feng Song, Xian-Shu Luo, Andy Eu-Jin Lim, Chao Li, Qing Fang, Tsung-Yang Liow, Lian-Xi Jia, Xiao-Guang Tu, Ying Huang, Hai-Feng Zhou, Guo-Qiang Lo

**Affiliations:** 1 Institute of Microelectronics (IME), Agency for Science, Technology and Research (A^*^STAR), Singapore, Singapore 117685; 2 State Key Laboratory on Integrated opto-electronics, College of Electronic Science and Engineering, Jilin University, Changchun 130012, China

## Abstract

Silicon photonics integrated circuits (Si-PIC) with well-established active and passive building elements are progressing towards large-scale commercialization in optical communications and high speed optical interconnects applications. However, current Si-PICs do not have memory capabilities, in particular, the non-volatile memory functionality for energy efficient data storage. Here, we propose an electrically programmable, multi-level non-volatile photonics memory cell (PMC) fabricated by standard complementary-metal-oxide-semiconductor (CMOS) compatible processes. A micro-ring resonator (MRR) was built using the PMC to optically read the memory states. Switching energy smaller than 20 pJ was achieved. Additionally, a MRR memory array was employed to demonstrate a four-bit memory read capacity. Theoretically, this can be increased up to ~400 times using a 100 nm free spectral range broadband light source. The fundamental concept of this design provides a route to eliminate the von Neumann bottleneck. The energy-efficient optical storage can complement on-chip optical interconnects for neutral networking, memory input/output interfaces and other computational intensive applications.

Computers have deeply impacted our daily lives in various fields and greatly improved the quality of life^1^. As technology progresses, more computational bandwidth is required at extremely high speeds, especially for supercomputers built from a cluster of core processors. Optical interconnection addresses the data bandwidth limitations faced by copper lines^2^, and silicon photonics have been a leading technology solution in this field^3^,^4^,^5^,^6^,^7^,^8^,^9^,^10^. Data storage or memory is complementary to high data transmission capability in maintaining a switched state, preferably using very little or no standby power. In essence, a non-volatile integrated optical memory on the PIC is a natural progression to enhance memory storage functionality for optical interconnects of the next paradigm in applications such as high performance computing^11^,^12^ and neural networking^13^.

There have been efforts in addressing integrated optical memory through the use of phase change material^14^,^15^,^16^, plasmonic memristor^16^,^17^, lasers^18^, nonlinear effect all-optical memory^19^,^20^ and Photoelectric conversion memory^21^,^22^. However, for process compatibility with the Si-PIC, a CMOS-compatible technology is generally preferred. Along this line, a silicon photonics with electrically erasable programmable read-only memory was suggested with simulated results to determine its feasibility^23^. Thus far, there has been no experimental demonstration of a memory block in a Si-PIC platform. This letter bridges the existing gap by proposing a photonics memory cell (PMC) for memory functionality in silicon photonics.

## Results

### Device designation and operation principles

**Figure 1:**
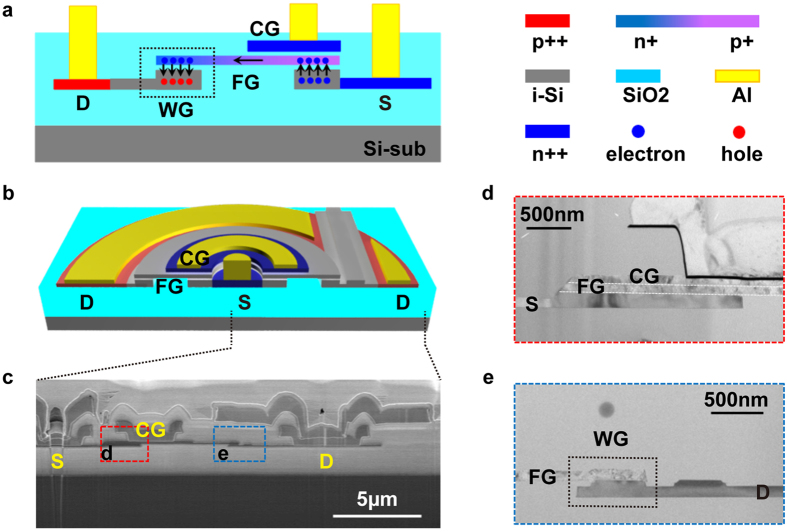
PMC operation principle and device structure. (**a**) Cross-sectional schematic of PMC showing the floating gate (FG) stack with separated n++ source (S) and p++ drain (D) configuration. The FG is doped n+ and p+ at the drain and source end, respectively. The control gate (CG) overlaps the FG partially at the source side. The FG stack at the drain is characteristic of a programmable MOS optical waveguide (WG). Black arrows denote the carrier movement during program and erase operation in a continuous flow. (**b**) Tilt schematic view of the MRR with the PMC and a ridge optical bus waveguide. (**c**) Cross-sectional SEM of the fabricated device. The radius of MRR is 10 μm, and both the MRR waveguide and bus waveguide widths were 500 nm. The slab thicknesses is 100 nm. The floating gate and control gate are 100 nm thick, either. (**d**) High resolution TEM image of the source region whereby the CG overlaps the FG, and (**e**) the drain region.

The cross-sectional image of the proposed Si-based PMC is illustrated in Fig. 1a. It comprises of an optical waveguide with a polycrystalline-silicon (poly-Si) floating gate, separated by a thin oxide dielectric in a metal-oxide-semiconductor (MOS) stack. Beneath the floating gate lies the drain and source terminals that are doped p- and n-type, respectively. At the source side, a poly-Si control gate overlaps the floating gate partially, and is separated from the floating gate by an oxide-nitride-oxide (ONO) stack.

The PMC is programmed by applying a positive voltage on the control gate, while the drain and source are grounded. This induces a high electrical field which injects electrons into the poly-Si floating gate through the tunneling oxide. The floating gate is doped in opposing polarity at the source and drain ends. This creates an internal electric field in the floating gate to assist the injected carriers to transverse from the source to the drain end when the bias on control gate is removed. Due to free carrier dispersion effect, the refractive index of the n-doped poly-Si is reduced^24^ and in turn, decreases the effective index of the waveguide core. The injected electrons are trapped within the floating gate after the bias is removed and creates a non-volatile memory state in the optical domain. The erase operation requires a positive voltage at the drain with the control gate and source grounded. This results in trapped electrons tunneling out into the drain from the floating gate. (Supplementary Fig. S1 shows the optical field distribution in the MOS waveguide, while Supplementary Fig. S2 shows the energy band diagrams for program and erase operation).

A bus waveguide-coupled microring resonator (MRR) was employed with the PMC to evaluate its memory functionality (See Methods for device fabrication details). The PMC is symmetrically radial in the MRR as seen in Fig. 1b. Figure 1c,e show the SEM and TEM cross section of the MRR. The MRR’s floating gate effective index is altered by biasing the appropriate device terminals which affects the optical spectrum carried by the bus waveguide.

### Demonstration of program/erase operation and non-volatile photonic memory property

**Figure 2:**
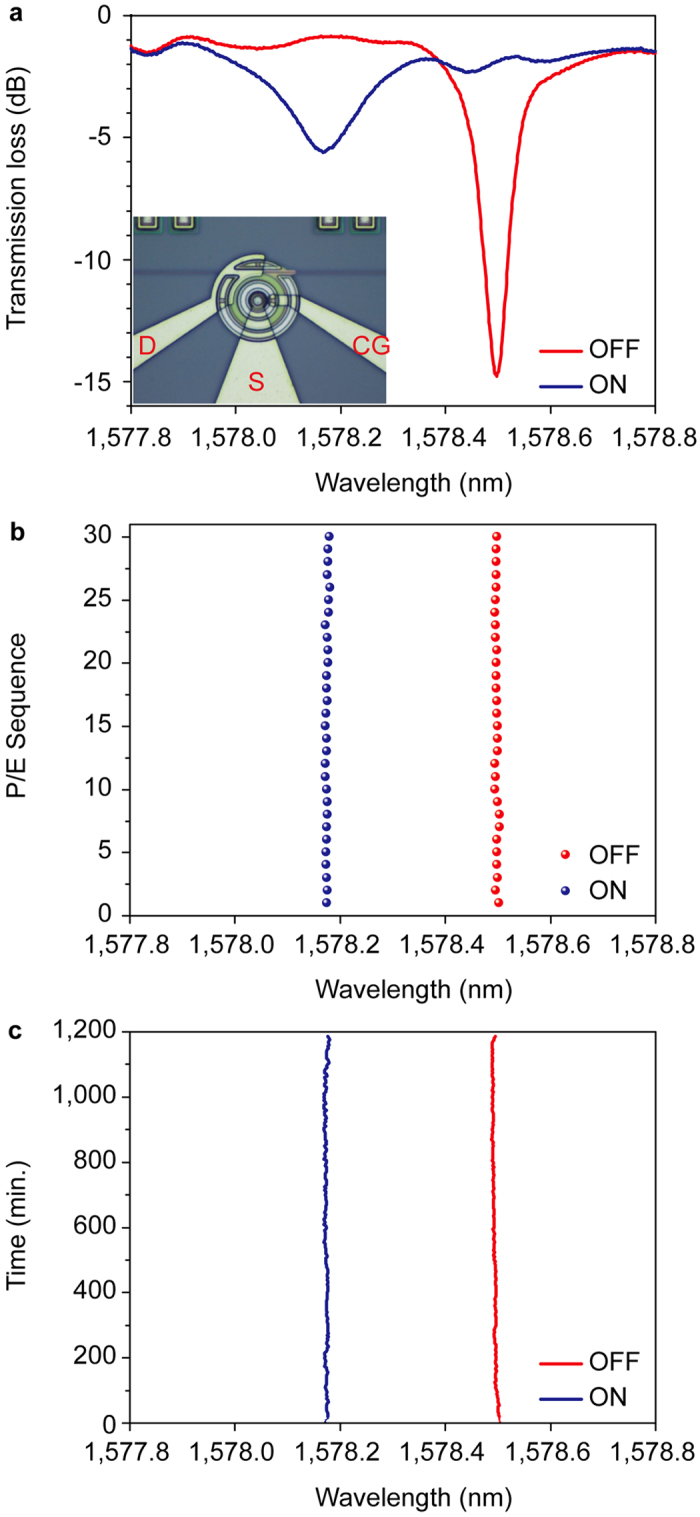
MRR non-volatile memory functionally. (**a**) The MRR transmission spectra at ON (blue) and OFF (red) states. Inset figure is microscopy picture of device. (**b**) The resonance wavelengths at ON and OFF states when the device is repeatedly programmed and erased for 30 sequential cycles. (**c**) The resonance wavelengths at ON and OFF states were unchanged within 20 hours.

The MRR was electrically programmed by sweeping the control gate voltage from 0 V to 20 V and back to 0 V, while grounding the source and drain. The injected electrons in the floating gate are transferred to the MRR waveguide at the drain side. A corresponding decrease in refractive index of the MRR waveguide results in a blue-shift of the resonant wavelength. This is labeled as the ON state. For the erasing process, the drain voltage is swept from 0 V to 6 V and back to 0 V with a grounded source and control gate. The MRR resonant wavelength red-shifts back due to electron extraction from the floating gate and this corresponds to the OFF state. Figure 2a shows the measured spectrum of the ON and OFF memory states. The wavelength difference of the ON and OFF states is ~325 pm. Q-factor of both of states are ~1.3 × 10^4^ (OFF) and ~9.6 × 10^3^ (ON), respectively.

The program and erase procedures were cycled 30 times for repeatability. The cumulative plots for the resonant wavelengths at ON and OFF states are summarized in Fig. 2b. A tight distribution with a standard deviation of σ~2.2 pm exhibits a stable performance under the stipulated test cycles. The optical spectrum was monitored for 20 hours for both ON/OFF states whereby the oscillation wavelength was traced every 5 min and showed in Fig. 2c. Although reliability stress tests were not conducted, it is believed that failure mechanisms are closely related to a similar flash memory transistor with the use of a traditional semiconductor-insulator-semiconductor gate stack and standard CMOS materials. An example of a reliability issue would be the generation of oxide traps and interface states at the tunnelling oxide after extended program and erase cycles. Nevertheless, the use of a relatively thick tunnel oxide of 8 nm minimizes trap-assisted electron tunnelling associated with this failure mechanism^25^.

### Multiple level photonic memory

**Figure 3:**
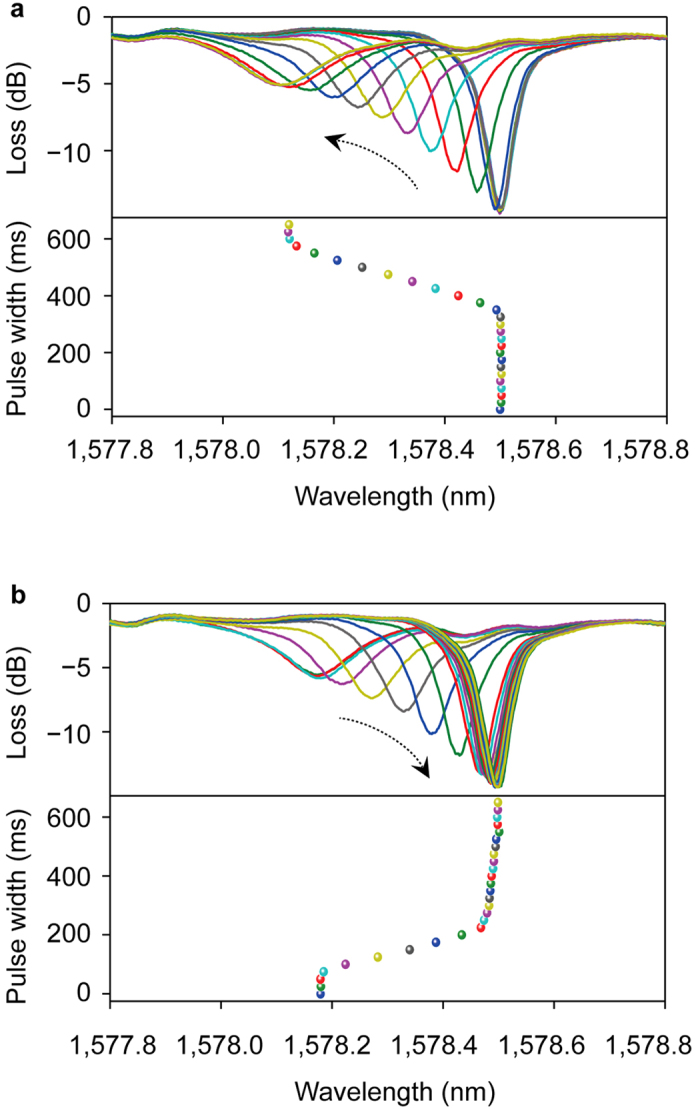
Investigation of MRR’s program and erase response. The resonance wavelength shifts after square-shaped pulse voltages with increasing pulse width are input to the device during (**a**) program (starting from OFF state) and (**b**) erase (starting from ON state). The pulse width increases from 0 to 625 ms with step of 25 ms.

Device programming capability was further investigated by supplying sequential square-shaped electrical pulses with peak voltage of 20 V at the OFF state. The pulse widths were increased sequentially from 0 ms to 625 ms, in steps of 25 ms, while monitoring the optical transmission spectrum. Figure 3a shows the optical spectra with increasing pulse widths, and plots the resonance wavelengths versus the corresponding pulse widths. The minimum pulse width to induce a blue shift was 350 ms which suggests that for pulse widths less than 350 ms, electrons were not injected into the floating gate. A maximum pulse width of 600 ms was required for a full resonance shift (~380 pm) with no additional resonance shifts observed when the pulse width was larger than 600 ms. The wavelength shift efficiency was ~1.66 pm/ms. Figure 3b shows the reverse operation simulating the erase step of the PMC. The peak voltage of the square-shaped signal is 6 V for erase. A pulse width of 225 ms and 75 ms, was required for minimum and maximum resonance shifts, respectively. The equivalent wavelength shift efficiency for erase was 1.96 pm/ms.

### Optical read-out memory array

**Figure 4:**
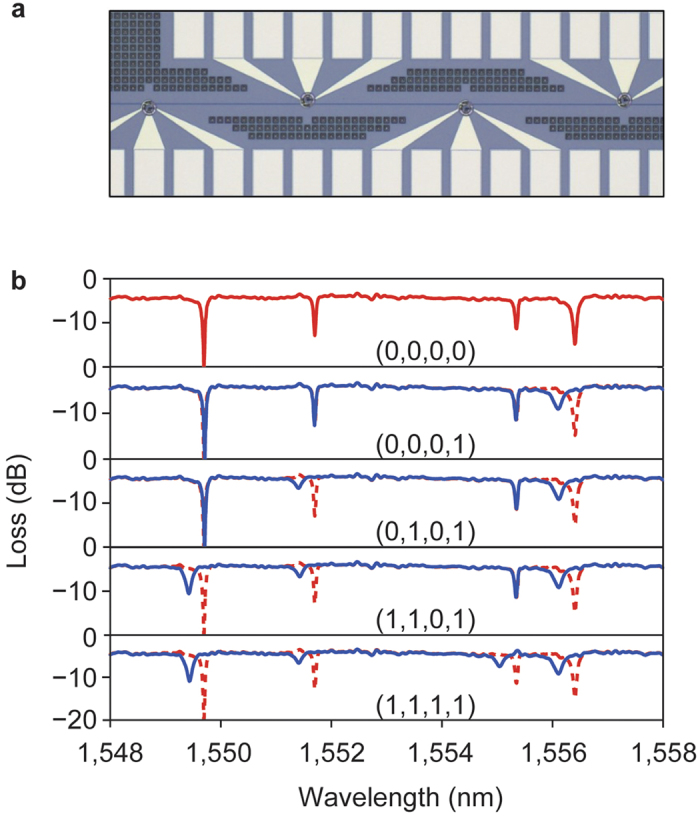
PMC applied as an optical readout memory array. (**a**) Optical microscope image of a four-channel MRR memory array coupled with a single bus waveguide. (**b**) Transmission spectra of different memory states when each of the MRRs are individually programmed. The (0, 0, 0, 0) memory state trace (in dotted RED) is superimposed onto subsequent plots for comparison to the different programmed 4-bit memory states.

The MRR was cascaded to form an optical read-out memory array. Unlike electrical readout where interference prevents reading multiple bits from the same bit line simultaneously, it is feasible to read multiple signals from an optical waveguide. To do so, different wavelengths are assigned to individual bits in a memory string and all bits can be read concurrently from the optical transmission spectrum. As a proof-of-concept, Fig. 4a shows a four-channel MRR memory array coupled to a single bus waveguide. The MRRs have slightly varying radii to couple different resonant wavelengths of the 4 bits. At the OFF state, all four MRRs have resonant wavelengths associated with a (0, 0, 0, 0) state, shown in the top plot in Fig. 4b. By selectively programming each of the MRR, different memory strings are realized through resonant wavelength changes as shown in the underlying plots.

The PMC can also be applied to Mach-Zehnder interferometer (MZI) structures which are much larger devices than MRRs. The change in memory states in the MZI arm controls the transmission output with extinction ratio of ~20–25 dB. Memory-functional MZI and MRR are basic structures, which can be extended to devices such as optical switches/routers, add-drop filters, and wavelength division multiplexers^26^,^27^. Supplementary Fig. S3, Fig. S4 and note S1 show the memory-functional MZI and add-drop MRR performance and discusses the fundamental applicability of the PMC in a wide range of photonic devices.

## Discussion

The effective refractive index of waveguide change Δ*n*_*eff*_ is obtained from:


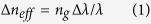


Where *n*_*g*_ is the group index (*n*_*g*_ is obtained from supplementary Fig. S5 and note S2), Δλ and λ is the change in wavelength and center wavelength respectively. The MRR waveguide effective index change is calculated to be 7.9 × 10^−4^. This gives a corresponding poly-Si floating gate refractive index change of ~3.1 × 10^−3^. From capacitance-voltage (*C-V*) measurements of the control gate and source, the measured voltage threshold window is 4.35 V at a capacitance of ~110 f F. This is close to the theoretical capacitance of ~133 f F. An estimation of the program and erase power consumptions are 17.2 pJ and 11.4 pJ, respectively. (A detailed discussion is given in supplementary Fig. S6 and note S3).

Power consumption can be reduced by decreasing the ON state wavelength shift from the OFF state and shrinking the MRR size. For example, the ON state wavelength shift be changed to 36 pm (the closest OFF state green line in Fig. 3a) and microring resonator’s radius shrinking to 5 μm. With those changes, the program and erase power consumptions can be improved to 0.476 pJ and 0.316 pJ, respectively.

Figure 3 shows that the PMC oscillation wavelength can be controlled by pulse width through the control gate, and hence realize multi-level memory capability^28^. There are two possible ways to achieve multi-level memory function using this device; firstly, by oscillation wavelength shift, and secondly, by amplitude change.

In the current program and erase process, the wavelength shift rates are 1.66 pm/ms and 1.96 pm/ms, respectively. This linear relationship allows control of the wavelength shift by modifying the electrical pulse width. If we determine the neighbor level spacing as 3σ (~6.5 pm), between ON and OFF states, even accommodate up to ~50 memory states. Obviously, a high Q-factor and a more stable system will contain more level states in optical memory cell. For the amplitude method, the input light wavelength is fixed. The OFF state oscillation wavelength of 1578.5 nm has a loss of ~14.7 dB as shown in Fig. 2a. As the oscillation wavelength blue-shifts, the loss becomes smaller. At final ON state, the optical loss is at its lowest of ~2 dB. In this transition, the different optical loss level can be defined as different memory level states. The utilization of multi-level memory will increase the memory capacity for data intensive storage.

As shown in Fig. 4, the PMC is employed in a multi-bit array. Assuming that a resonance FWHM of ~117 pm gives an optical channel spacing of ~234 pm (two times of FWHM), a MRR memory array with a 10 nm free spectral range can pack up to ~40 bits. Therefore, for a 100 nm broadband light source, a memory read capacity of up to 400 times faster is possible over traditional electrical memory read. This property is expected to overcome the von Neumann bottleneck which is caused by the data exchange speed limitation between processors and memory.

In summary, we have experimentally demonstrated the first prototype of a CMOS platform compatible, electrical programmable, non-volatile multi-level photonic memory cell in Si-PICs. A MRR structure was employed to experimentally demonstrate the PMC’s operation and usability. The increase in memory reading speed and data throughput was demonstrated as well. The PMC design shows potential in overcoming the von Neumann bottleneck to be used in advanced computer and neural networks^29^.

## Methods

### Fabrication process

The devices was fabricated in IME/A*STAR’s 8-inch semiconductor processing line. Silicon-on-insulator (SOI) wafer with 160 nm top silicon on 2 μm buried oxide (BOX) was used as the starting substrate. A 500 nm rib waveguide with 100 nm slab thickness was formed on BOX. Inverse Si tapers were used as input and output couplers. Oxide cladding was deposited and planarized to leave a thin oxide of 100 nm above the waveguide. A plasma etch process was employed to remove the thin oxide and expose the top silicon for thermal oxidation. 8 nm of oxide was thermally grown to be used as the gate oxide. These process steps allowed precise gate oxide thickness control without damaging the Si waveguide. Subsequently, 100 nm of amorphous silicon was deposited by low pressure chemical vapor deposition (LPCVD), followed by phosphorus and boron implantation (3 × 10^12^ cm^−2^, 13 keV). A film stack consisting of 6 nm tetraethyl orthosilicate (TEOS) oxide, 3.5 nm LPCVD Si_3_N_4_, and 6 nm TEOS oxide was deposited to form the oxide-nitride-oxide (ONO) layer. Finally, a 100 nm LPCVD poly-silicon was deposited to complete the device structure. To form the electrical contacts, the poly-silicon, ONO, and cladding oxide layers on the waveguide slab was etched away for implantation. In order to protect the floating gate sidewall, a SiO_2_/SiN spacer was formed. High dose phosphorus (4 × 10^15^ cm^−2^, 35 keV) and boron (5 × 10^14^ cm^−2^, 13 keV) implant was to form source/gate, and drain, respectively. After dopant activation anneal, 6 kA SiO_2_ was deposit and contact holes were etched, followed with a two-level Al metallization.

### Testing setup

A 2.5 μm lensed fiber was used to couple light into the PIC. Broadband amplified spontaneous emission (ASE) laser (EXFO FLS-2300B) was used as input light source. An Agilent 8169 A polarization controller was used to select transverse electric (TE) polarization for the input light. Using the waveguide cutback method, channel waveguide loss of 2.6 dB/cm was extracted. The fiber/waveguide coupling loss is ~3.4 dB/facet, and total insertion loss is ~8.6 dB for MRR PMC structure. An ANDO AQ6317B was used as the optical spectrum analyzer. The optical resolution was set at 10 pm. An Agilent B1500A semiconductor device analyzer was used as the electrical power supply and the memory program, memory erase, and pulse electrical signals. To avoid excessive current to the device, current compliance was set at 10 nA. Hewlett Packard Precision LCR Meter (4284 A) was used for the *C-V* measurements.

## Additional Information

**How to cite this article**: Song, J.-F. *et al.* Integrated photonics with programmable non-volatile memory. *Sci. Rep.* **6**, 22616; doi: 10.1038/srep22616 (2016).

## Supplementary Material

Supplementary Information
