# Supplementary material for: Integrated photonics with programmable non-volatile memory
Source: Sci Rep. 2016 Mar 4;6:22616. doi: 10.1038/srep22616 (PMC4778119; doi:10.1038/srep22616)
Supplement: Supplementary Information [file srep22616-s1.pdf]

Integrated photonics with programmable non-volatile memory

-Supplementary information

Jun-Feng Song[1,2]*, Xian-Shu Luo[1], Andy Eu-Jin Lim[1], Chao Li[1], Qing Fang[1], Tsung-Yang Liow[1], Lian-Xi Jia[1], Xiao-Guang Tu[1], Ying Huang[1], Hai-Feng Zhou[1] & Guo-Qiang Lo[1]

[1]Institute of Microelectronics (IME), Agency for Science, Technology and Research (A*STAR), Singapore, Singapore 117685

[2]State Key Laboratory on Integrated opto-electronics, College of Electronic Science and Engineering, Jilin University, Changchun 130012, China

*e-mail: songjf@ime.a-star.edu.sg

Supplementary figures

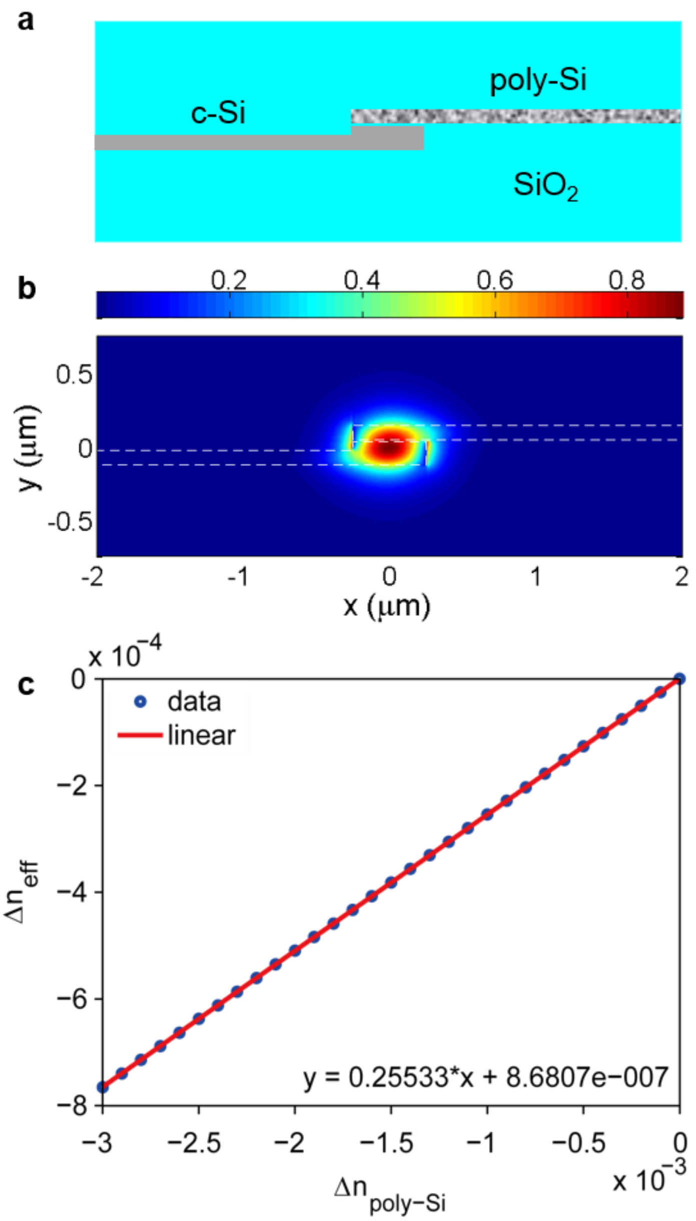

Supplementary Fig. S1 Simulation results of MOS waveguide. a. Schematic of MOS waveguide. The thicknesses of the crystalline silicon (c-Si) slab and poly-silicon (poly-Si) are 100 nm. The center c-Si is 160 nm thick and 500 nm width. The tunneling gate oxide is 8 nm. **b**. TE mode field distribution of the MOS waveguide whereby strong optical mode overlap with the floating gate is

seen. The white dished line shows the c-Si and poly-Si waveguide profiles. **c.** The effective index change of the MOS waveguide with poly-silicon floating gate's refractive index variation. The blue circle markers are calculated data, while the red line is a linear fit with a slope of ~0.255.

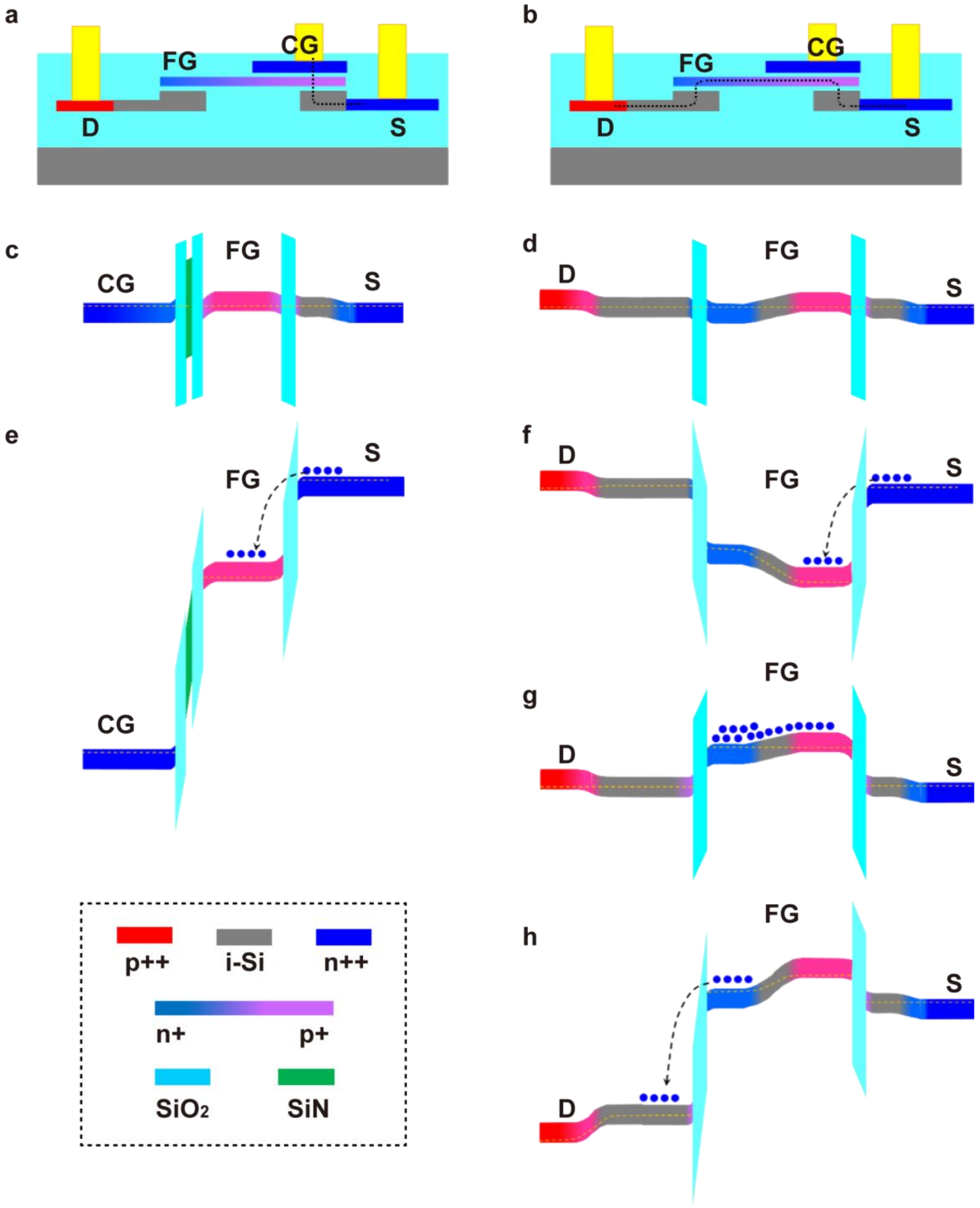

Supplementary Fig. S2 Energy band diagrams during program and erase. **a.** and **b.** The schematic of the PMC cross section structures. Dotted black lines are positions for tracing energy band in program and erase. The doped poly-Si floating gate has a *pn* junction between the source and the drain to assist

carriers in drifting to the drain end. The control gate and source are highly n-doped terminals. An oxide-nitride-oxide (ONO) stack (6nm SiO_2/3.5 nm SiN/6 nm SiO_2) lies in between the control gate and the floating gate, and an 8nm tunneling oxide separates the control gate from the source. **c.** and **d.** The energy band diagrams for the OFF states. **e.** and **f.** The energy band diagrams during program operation. For the program (write) operation, the control gate is biased with a voltage and the dielectric energy bands begin to bend. At sufficiently high voltage, electrons tunnel through the reduced energy barrier of the tunneling oxide via Fowler-Nordheim (FN) tunneling to achieve the floating gate. The ONO stack restricts electrons from tunneling to the control gate by trapping electrons in the SiN layer because of the band gap discontinuity between SiN and SiO_2. **g**. The energy band diagram for the ON state. After bias at the control gate is removed, the injected electrons are redistributed from the source side to the drain side due to the presence of the PN junction. The PMC is in the ON state. **h**. The energy band diagram during erase operation. For the erase operation, the drain is biased with a voltage. At sufficiently high voltage, electrons tunnel through the decreased energy barrier of the tunneling oxide again.

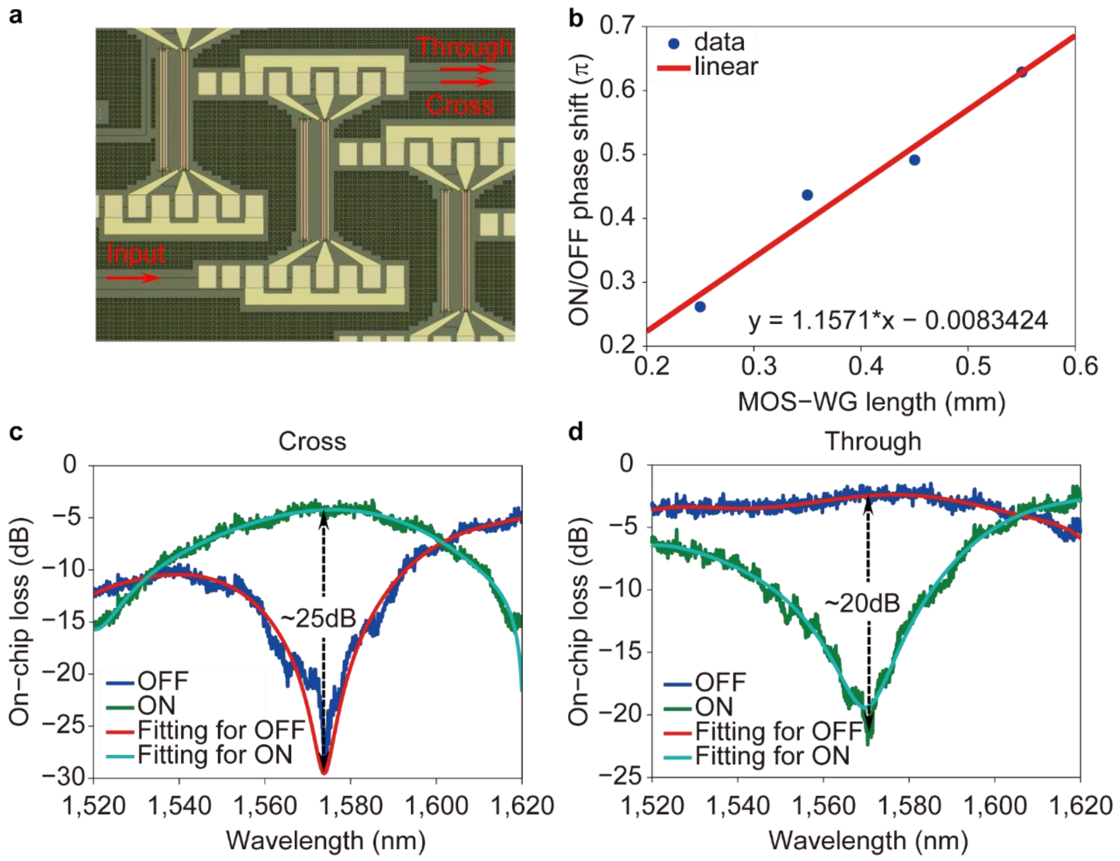

Supplementary Fig. S3 Memory-functionalized MZI structure. a. An optical microscope of 2 × 2 MZI structure. **b.** ON/OFF phase shift versus WG length. **c.** Transmission spectrum for cross port in ON and OFF states. **d.** Transmission spectrum for the corresponding through port in ON and OFF states.

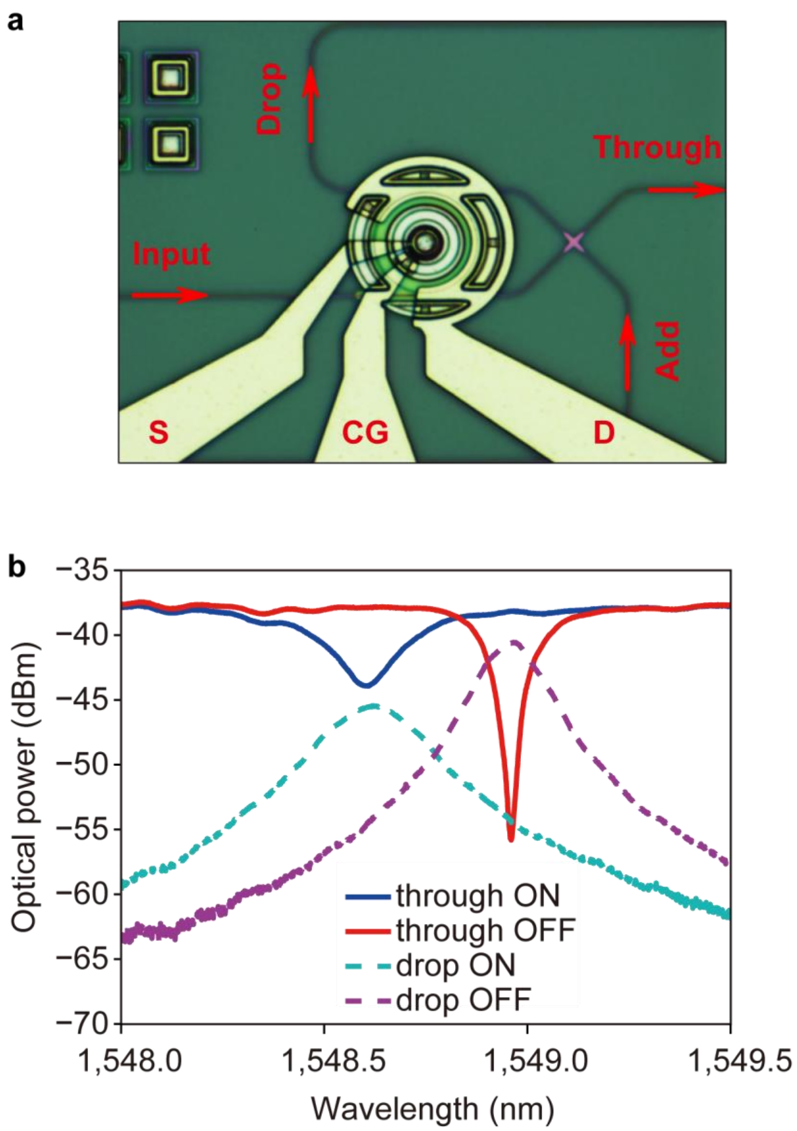

Supplementary Fig. S4 Add/drop memory functionalized MRR. a. Optical microscope image of the add/drop MRR. The MRR radius is 15 μm. **b.** ON/OFF transmission spectrum of through and drop ports.

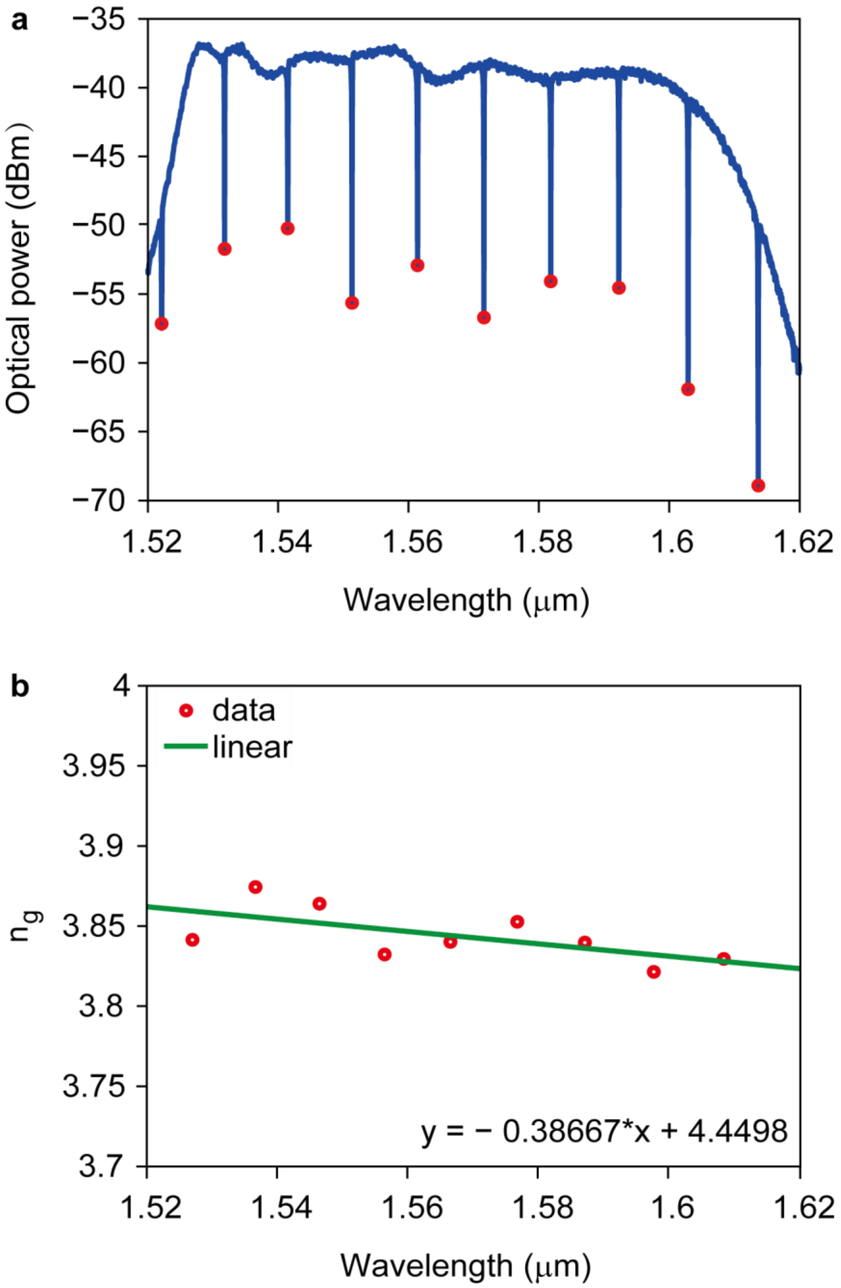

Supplementary Fig. S5 Optical spectrum and effective index of the MRR.

a. Transmission spectrum of a 10 μm radius MRR with a free spectral range (FSR) of 10 nm. The red circles denote the oscillation peaks. **b.** Group index was calculated by Eq. (N1-2) and plotted (red circles). The green line is the linear fit.

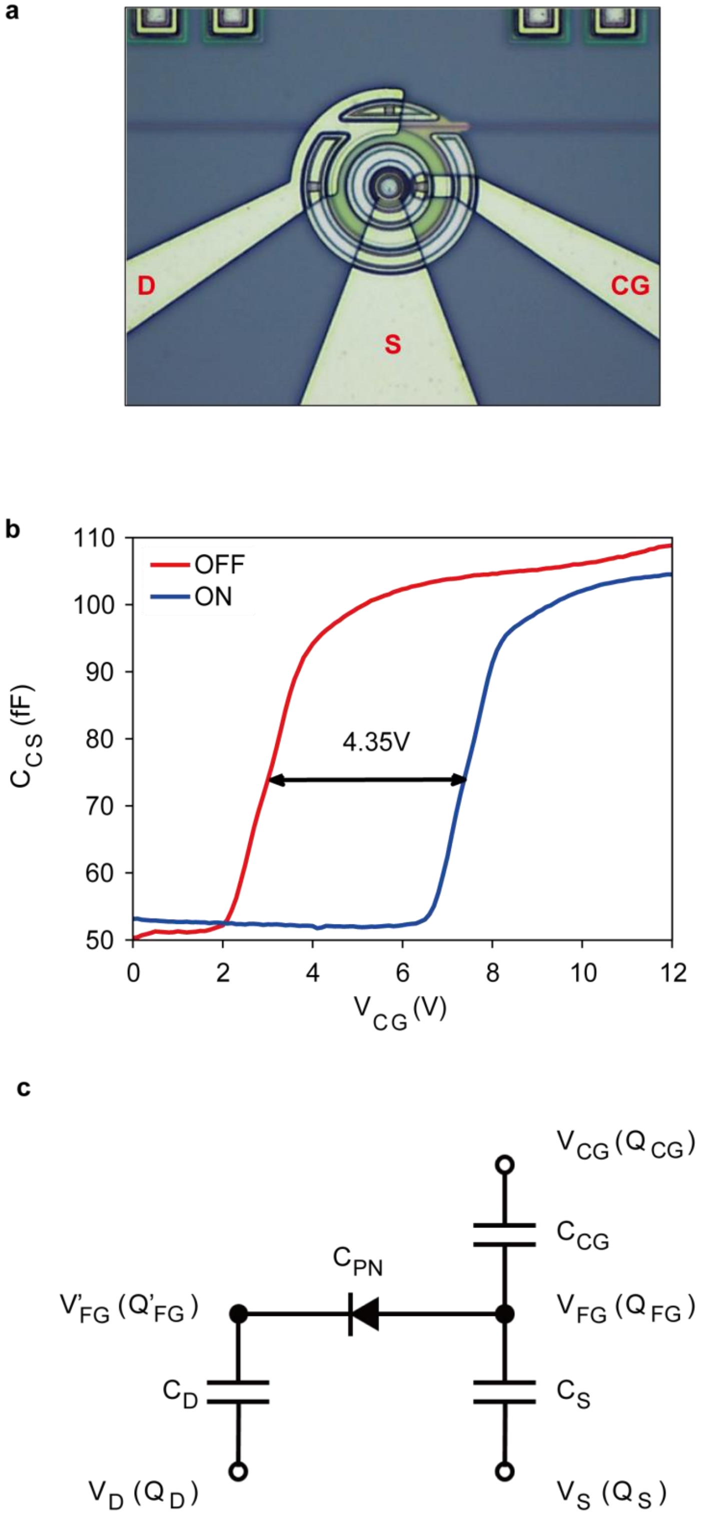

Supplementary Fig. S6 Electrical properties of the MRR. a. An optical microscope image of the MRR. The drain (D), source (S), and control gate (CG)

are labeled as terminals. **b.** Capacitance-voltage (*C-V*) curves between the control gate and source during ON (blue line) and OFF state (red line). The measured *C-V* with a threshold voltage window of 4.35 V between the ON and OFF states. The source terminal was grounded at 0 V and the drain terminal was floated when the control gate was swept for measurement. The control gate voltage was swept from 0 V to 12 V in steps of 100 mV. **c.** An illustration of the device capacitance.

Supplementary notes

Supplementary note S1 Non-volatile memory functional Mach-Zehnder interferometer (MZI) and add/drop MRR

MZI and MRR are essential elements to build optical WDM systems for e.g. 2×2 MZI and add/drop MRR (AD-MRR). 2×2 MZI matrix are used for non-block optical switching and optical cross connect (OXC)[1, 2], while AD-MRRs are employed in low power consumption optical router[3, 4]. Furthermore, these can be applied into larger systems such as wavelength selective switch (WSS) and reconfigurable optical add/drop multiplexer (ROADM). By integration memory functionality into MZI and AD-MRR, we can build up on-chip optical networks that will greatly enrich optical networking capabilities and reduce power consumption.

Memory-functionalized MZI is made of two 2 × 2 directional couplers and integrated with the PMC in both arms. The directional coupler is built using two 40 μm channel waveguides and a gap of 300 nm. Two arms of the channel waveguide have a length difference of 25 μm to induce a phase difference. The microscope picture is shown in **Fig. S3a**. **Fig. S3b** plots the ON/OFF phase shift versus the MOS-WG length. Through linear fitting, a slope of 1.16 π/mm is extracted. Therefore, a π phase shift requires 865 μm in MOS-WG length. **Fig. S3c** and **S3d** indicate the transmission spectrum changes in the cross and through port during ON and OFF states. Extinction ratios of ~25 dB and ~20 dB were achieved during ON and OFF states.

An all-pass MRR was described in the main manuscript. Using the MRR, an

add/drop MRR optical switch can be alternatively constructed. **Fig. S4a** shows the optical microscope image of the add/drop MRR switch where add and drop channels are indicated. The transmission spectrum of the two output ports (through and drop) at the different memory states are shown in **Fig. S4b.** At a wavelength of 1548.96 nm, the device is at an OFF state. The extinction ratio of through and cross output is 18 dB and 16 dB, respectively.

Supplementary note S2 Dispersion relationship of MOS waveguide

Fig. S2a plot the spectrum of the MRR. The red color circles denote oscillation peaks, λ_j. The relationship of the oscillation wavelength with effective index is given in Eq. (N2-1). Using group index definition as $n_g = n_{eff} - \lambda \frac{\partial n_{eff}}{\partial \lambda}$, Eq. (N2-2) is derived.

$$\begin{cases} n_{eff}\left(\lambda_{m-1}\right)L = \left(m-1\right)\lambda_{m-1} \\ n_{eff}\left(\lambda_{m}\right)L = m\lambda_{m} \\ n_{eff}\left(\lambda_{m+1}\right)L = \left(m+1\right)\lambda_{m+1} \end{cases} \tag{N2-1}$$

$$\frac{1}{n_g\left(\lambda_m\right)L} = \frac{1}{2}\left(\frac{1}{\lambda_{m+1}} - \frac{1}{\lambda_{m-1}}\right) \tag{N2-2}$$

Where, n_{eff} is effective index of MOS waveguide, L is the perimeter of the MRR, m is an integer, and λ_m is the oscillation wavelength.

The red dotted data in **Fig. S2b** were calculated by Eq. (N2-2) and the green line is the linear fit.

For carriers injected into MOS waveguide, the oscillation wavelength is shifted as described in Eq. (N2-3). This is because MOS waveguide effective index has changed.

The MOS waveguide effective index change is affected by two parts; firstly, carrier-induced and secondly, wavelength shift (dispersion)-induced. These are described in Eq. (N2-4). Considering Eq. (N2-1), and the wavelength dispersion effect, we can get Eq. (N2-5). By replacing n_g in Eq. (N2-5), the relationship of resonant wavelength and effective index change by carrier's dispersion can be expressed in Eq. (N2-6).

$$Ln'_{eff}\left(\lambda_m+\Delta\lambda\right)=m\left(\lambda_m+\Delta\lambda\right) \tag{N2-3}$$

$$\Delta n_{eff}=n'_{eff}-n_{eff}=\left[\Delta n_{eff}\right]_N+\left[\Delta n_{eff}\right]_\lambda \tag{N2-4}$$

$$\begin{cases}\Delta n_{eff}=\dfrac{m}{L}\Delta\lambda_m=n_{eff}\left(\lambda_m\right)\dfrac{\Delta\lambda_m}{\lambda_m}\\ \left[\Delta n_{eff}\right]_\lambda=\dfrac{\partial n_{eff}}{\partial\lambda}\Delta\lambda\end{cases} \tag{N2-5}$$

$$\left[\Delta n_{eff}\right]_N=\Delta n_{eff}-\left[\Delta n_{eff}\right]_\lambda=\frac{\Delta\lambda}{\lambda}n_g \tag{N2-6}$$

Supplementary note S3 Power consumption of MRR

For program operation, the PN junction is forward conduction state. We assume it is equivalent to the conductor. From the conservation of charge in a capacitor:

$$Q_{FG}+Q_{CG}+Q_S+Q_D=0 \tag{N3-1}$$

Where Q_{FG} is the charge at the floating gate terminal, Q_{CG} is the charge at the control gate terminal, Q_D is the charge at the drain and Q_S is the charge at the source. Q_{CG}, Q_S and Q_D can be represented as:

$$\begin{cases}Q_{CG}=C_{CG}\left(V_{CG}-V_{FG}\right)\\ Q_D=C_D\left(V_D-V_{FG}\right)\\ Q_S=C_S\left(V_S-V_{FG}\right)\end{cases} \tag{N3-2}$$

Where C_{CG} is the capacitance between the control and floating gate, C_D is the

capacitance between the floating gate and drain, and C_S is the capacitance between the floating gate and source. The voltages of the control gate, floating gate and source are expressed as V_{CG}, V_{FG} and V_S, respectively. Replacing Eq. (N3-2) into Eq. (N3-1), Eq. (N3-3) is derived to be:

$$Q_{FG} + C_{CG}\left(V_{CG} - V_{FG}\right) + C_D\left(V_D - V_{FG}\right) + C_S\left(V_S - V_{FG}\right) = 0 \qquad \text{(N3-3)}$$

Finally, V_{FG} can be expressed as:

$$V_{FG} = \frac{Q_{FG} + C_{CG}V_{CG} + C_D V_D + C_S V_S}{C_{CG} + C_D + C_S} \qquad \text{(N3-4)}$$

A transition from OFF state to ON state increases the floating gate charge from 0 to Q_{FG}. When charges are injected from source to floating gate, power consumption during program, W_{prog} is calculated using:

$$W_{prog} = -\int_0^{Q_{FG}} V_{FG}\,dQ = -\frac{1}{2}\frac{Q_{FG}^2}{C_{CG} + C_D + C_S} - \frac{Q_{FG}\left(C_{CG}V_{CG} + C_D V_D + C_S V_S\right)}{C_{CG} + C_D + C_S} \qquad \text{(N3-5)}$$

The negative sign indicates that current is in reverse direction. Q_{FG} is determined by

$$Q_{FG} = -C_{CG}\Delta V_T \qquad \text{(N3-6)}$$

Where ΔV_T is threshold window. ΔV_T is shown to be 4.35 V in **Fig. S6b.**

For erase operation, the PN junction is in backward state. The capacitor is very small. So we assume $C_{PN} \cong 0$ and $Q'_{FG} = Q_{FG}$. Similarly, the power consumption during erase, W_{erase} is given as:

$$W_{erase} = -\int_{Q'_{FG}}^{0} \left(V'_{FG} - V_D\right) dQ = \frac{1}{2}\frac{Q'^2_{FG}}{C_D} \qquad \text{(N3-7)}$$

Where C_D is capacitance between floating gate and drain.

For the device in **Fig. S6a**, the control gate area is a disk with an outer and inner radius

of 7.7 µm and 2.74 µm, respectively. With an ONO dielectric stack, the calculated C_{CG} is 404.3 fF using a relative permittivity of 3.9 and 7.0 and SiO_2 and SiN, respectively. C_{FS} is also calculated to be 197.7 fF, and C_D is 135.6 fF.

Supplementary Reference

1 Papadimitriou, G. I., Papazoglou, C. and Pomportsis, A. S. Optical switching: switch fabrics, techniques, and architectures. *J. Lightwave Technol.* **21,** 384-405 (2003).

2 Yang, M. *et al.*, Non-blocking 4x4 electro-optic silicon switch for on-chip photonic networks. *Opt. Express* **19,** 47-54 (2011).

3 Nikolova, D. *et al.*, Scaling silicon photonic switch fabrics for data center interconnection networks. *Opt. Express* **23,** 1159-1175 (2015).

4 Sherwood-Droz, N. *et al.*, Optical 4x4 hitless slicon router for optical networks-on-chip (NoC). *Opt. Express* **16,** 15915-15922 (2008).
